# Establishment of a Simple, Sensitive, and Specific *Salmonella* Detection Method Based on Recombinase-Aided Amplification Combined with dsDNA-Specific Nucleases

**DOI:** 10.3390/foods13091380

**Published:** 2024-04-29

**Authors:** Changyu Zhou, Yu Zhao, Boyan Guo, Ming Yang, Qiang Xu, Changwei Lei, Hongning Wang

**Affiliations:** 1 Key Laboratory of Bio-Resource and Eco-Environment of Ministry of Education, College of Life Sciences, Sichuan University, Chengdu 610017, China; l1z2l3y4@163.com (C.Z.); zhaoyu3@stu.scu.edu.cn (Y.Z.); gby0229@163.com (B.G.);; 2 Animal Disease Prevention and Food Safety Key Laboratory of Sichuan Province, Chengdu 610064, China

**Keywords:** dsDNase, one pot, RAA, *Salmonella*, visualization

## Abstract

*Salmonella* is a common foodborne pathogen that can cause food poisoning, posing a serious threat to human health. Therefore, quickly, sensitively, and accurately detecting *Salmonella* is crucial to ensuring food safety. For the *Salmonella hilA* gene, we designed Recombinase-aided amplification (RAA) primers and dsDNA-specific nuclease (DNase) probes. The ideal primer and probe combination was found when conditions were optimized. Under UV light, a visual *Salmonella* detection technique (RAA-dsDNase) was developed. Additionally, the RAA-dsDNase was modified to further reduce pollution hazards and simplify operations. One-pot RAA-dsDNase-UV or one-pot RAA-dsDNase-LFD was developed as a *Salmonella* detection method, using UV or a lateral flow dipstick (LFD) for result observation. Among them, one-pot RAA-dsDNase and one-pot RAA-dsDNase-LFD had detection times of 50 min and 60 min, respectively, for detecting *Salmonella* genomic DNA. One-pot RAA-dsDNase-UV had a detection limit of 10^1^ copies/μL and 10^1^ CFU/mL, while one-pot RAA-dsDNase-LFD had a sensitivity of 10^2^ copies/μL and 10^2^ CFU/mL. One-pot RAA-dsDNase-UV and one-pot RAA-dsDNase-LFD assays may identify 17 specific *Salmonella* serovars witho ut causing a cross-reaction with the remaining 8 bacteria, which include *E. coli*. Furthermore, *Salmonella* in tissue and milk samples has been reliably detected using both approaches. Overall, the detection method developed in this study can quickly, sensitively, and accurately detect *Salmonella*, and it is expected to become an important detection tool for the prevention and control of *Salmonella* in the future.

## 1. Introduction

Foodborne pathogen-induced infectious illnesses are considered a major global public health concern, posing serious risks to human and animal health and resulting in large financial losses [1,2,3]. *Salmonella* strains are considered the most common foodborne pathogen globally; they are primarily responsible for foodborne disease outbreaks and infections due to their natural environment frequency [4] and stability in food production and retail supply chains [5]. *Salmonella* is categorized as a moderate-to-serious risk pathogen by the World Health Organization [6], and it is estimated that it has resulted in over 80 million illnesses to date [7]. Thus, a quick, extremely sensitive, and easy-to-use detection technique is desperately needed to stop the spread of *Salmonella*.

For diagnosing *Salmonella*, conventional laboratory culture techniques were regarded as the “gold standard”. Unfortunately, those methods were extremely complicated and require several processes, including pre-enrichment, bacterial multiplication, and selective separation [8,9]. As a result, it used to take more than 3 days to obtain the desired results [10]. *Salmonella* detection has undergone a revolution with the development of nucleic acid amplification detection (NAAT) technology. NAAT-based techniques, such as polymerase chain reaction (PCR) [11], real-time quantitative PCR (qPCR) [12], and CRISPR/Cas13a [13] are now frequently used for *Salmonella* detection. Nevertheless, these techniques have intrinsic drawbacks that restrict the range of situations in which they can be used, such as the demand for costly machinery, strict environmental regulations, and skilled operators [14]. Moreover, loop-mediated isothermal amplification (LAMP) has been extensively utilized in the detection of *Salmonella*, although it necessitates an intricate primer design [15,16].

Recombinase-aided amplification (RAA) has a number of benefits, such as rapidity, low cost, high sensitivity, and the capacity to quickly amplify DNA at low temperatures (37–42 °C). In recent years, these characteristics have rendered RAA technology appropriate for the identification of viruses and bacteria [17,18]. Nevertheless, this method frequently calls for the addition of agarose gel electrophoresis, and even the RAA instruction manual states that the purification of RAA products is required prior to electrophoresis, which considerably reduces the sensitivity and speed of RAA detection. Numerous investigations have exhibited the possibility of utilizing RAA in conjunction with the CRISPR/Cas system to optimize detection sensitivity and efficiency while also accelerating the procedure [19,20]. However, the CRISPR/Cas technique usually necessitates pre-preparing crRNA and places more demands on the environment used for detection. As a result, the practical implementation of RAA in conjunction with other approaches is highly valuable for *Salmonella* identification.

Highly selective endonucleases, known as double-stranded DNA-specific nucleases (dsDNase), cleave phosphodiester bonds in double-stranded DNA to produce oligonucleotides with 3′-hydroxyl and 5′-phosphate termini. These enzymes cannot break down single-stranded DNA or RNA, but they are highly selective for double-stranded DNA [21]. In one investigation, the application of dsDNase was used to detect miRNA-10b and enhance the signal strength produced during the reaction [22]. As far as we are aware, no reports exist about RAA-dsDNAse target detection.

To identify *Salmonella* without requiring RNA in the reaction, we used dsDNase instead of CRISPR/Cas in this study and paired it with RAA, which simplified the environmental conditions needed for detection. The effectiveness of this technique depends on the choice of a suitable target for detection. Based on the information at hand, a number of genes, including invA, stn, opmC, fimA, iroB, agfA, and fimA, have been used to identify *Salmonella*. Nevertheless, some of these genes are absent from particular *Salmonella* strains, which reduces their specificity and limits the range of situations in which they can be used. According to the research, all invasive strains of *Salmonella* include the *Salmonella* Pathogenic Island 1 gene (SPI1), which is essential for *Salmonella* invasion. One important virulence component in *Salmonella* invasion and infection is SPI1. Among the positively transcribed regulatory genes encoded in SPI1 is the *hilA* gene [23,24]. Because it influences *Salmonella* colonization, the *hilA* gene is essential for the pathogenesis of *Salmonella*. The *hilA* gene is extremely unique to *Salmonella* and has not been found in any other Gram-negative bacteria [25]. Using PCR or LAMP techniques that target the *hilA* gene, some researchers have successfully developed *Salmonella* detection methods [26]. This implies that concentrating research efforts on the *hilA* gene is reasonable.

Combining RAA with dsDNase allowed us to create a novel *Salmonella* detection technique called RAA-dsDNase. One-pot detection refers to a simplified analytical approach that allows for the simultaneous detection and quantification of multiple analytes in a single reaction vessel, eliminating the need for separate sample preparation and multiple reaction steps. So, we developed the one-pot RAA-dsDNase-UV and one-pot RAA-dsDNase-LFD procedures by placing an RAA and DNase reaction in a tube and observing the outcomes with UV and LFD.

## 2. Methods and Materials

### 2.1. Reagents and Materials

The primers and fluorescent probes used in this study were purified by high-performance liquid chromatography (HPLC) and synthesized by Sangon Biotechnology Co., Ltd. (Shanghai, China). The 2 X M5 HiPer Taq HiFi PCR mix (with blue dye) was purchased from Mei5 Biotechnology Co., Ltd. (Beijing, China). The supplier of the RAA kit was Hangzhou ZC Bio-Sci & Tech Co., Ltd. (Hangzhou, China). We bought the dsDNase from Yeasen Biotechnology Co., Ltd. (Shanghai, China) along with an RNase inhibitor. The supplier of the tissue DNA extraction kits and bacterial genomic DNA extraction kits was Tiangen Biochemical Technology Co., Ltd. (Beijing, China).

### 2.2. Bacterial Preparation and DNA Extraction

The bacteria used in this investigation were maintained in a freezer at −20 °C with 50% glycerol. Before being used, the bacteria were allowed to grow on Luria-Bertani (LB) media plates. A single colony was picked and transferred to LB liquid medium and grown in an incubator until the OD_600_ reached 0.8. At this stage, bacterial cells were used to extract DNA using a bacterial genomic DNA extraction kit.

### 2.3. Construction of Target Gene pMD19-T-hilA Vector

The *hilA* gene was PCR-amplified, separated on agarose gel electrophoresis, and the product was recovered. This insert was ligated to the pMD19-T vector to generate the pMD19-T-*hilA* construct (Figure S1).

### 2.4. Design and Screening of RAA Primers and dsDNAse Probes

To create the RAA primers and dsDNAse probes, a highly conserved gene sequence was chosen by retrieving and evaluating the conservation of the *hilA* gene in the NCBI database. In all, nine primer and two probe pairs were created, and primer and probe screening was performed, utilizing the *hilA* gene-containing plasmid as a template. The RAA reaction system was set up as follows: the RAA reaction dry powder tube was filled with 25 μL of A Buffer and 13.5 μL of ddH_2_O. After thorough mixing, the mixture was then evenly divided into five new Eppendorf (EP) tubes. Following this, 1 μL of the template and 0.4 μL of each of the downstream and upstream primers (10 μM) were added to each EP tube. Lastly, each EP tube cap was filled with 0.5 μL of B buffer, making a total reaction volume of 10 μL. The mixture was covered, gently inverted eight to ten times, and then centrifuged for 10 s at a low speed. For 40 min, the reactions were conducted at 37 °C. After the reaction, 2% agar gel electrophoresis was used to examine the RAA products, and the bands were observed under UV light. Primer combinations with bright, single bands were selected for additional testing. To make a total volume of 20 μL, the dsDNase reaction system was supplemented with water. It included 10 μL RAA products, 2 μL of 10× dsDNase buffer, 1 μL of probe (10 μM), 1 μL of dsDNAse, 2 μL of template DNA, or ddH_2_O. The reaction mixture was incubated for 10 min at 37 °C, and the outcomes were examined under UV light.

### 2.5. Establishment of RAA-dsDNase Detection Method

Primers F7R7 and probe-2 were used to create the RAA-dsDNase detection method. The reaction system and procedure were identical to those outlined above. As negative controls, the dsDNase reaction system without adding dsDNase and the RAA reaction system without adding the DNA template were employed.

### 2.6. RAA-dsDNase Reaction Condition Optimization

The RAA reaction system is completed prior to the degradation reaction of dsDNase and its reaction conditions are almost optimal. Hence, the primary goal was to optimize the conditions for the dsDNase degradation reaction. Unless otherwise noted, dsDNase had a 10 min reaction time. For optimizing the reaction buffer, we added 0 μL, 0.5 μL, and 1 μL of 10× dsDNase buffer to the dsDNase reaction system and assessed the impact of the amount of the buffer on the degradation ability of RAA-dsDNase. Dosage optimization for the dsDNase enzyme is required since the concentration of the enzyme directly influences its capacity for breakdown. Therefore, we introduced 0.5 μL, 0.75 μL, and 1 μL of dsDNase to the reaction systems. Each reaction system’s fluorescence intensity was measured under UV light after it had been reacting for 10 min at 37 °C. Reaction time optimization: Due to dsDNase’s own high capacity for target degradation, we measured the fluorescence intensity in the reaction system at 0, 5, and 10 min of the dsDNase reaction, respectively.

### 2.7. Sensitivity and Specificity Analysis of RAA-dsDNase and PCR Specificity Analysis

After the optimization of conditions, we conducted a sensitivity and specificity analysis of RAA-dsDNase. We used a continuous 10-fold dilution method, with pMD19-T-*hilA* plasmid and *Salmonella typhimurium* (ATCC14028) genomic DNA as templates, for the RAA-dsDNase sensitivity analysis. Additionally, the genomic DNA of eight additional bacterial species, including *E. coli*, and 17 serovars of *Salmonella* were utilized as templates for RAA-dsDNase and PCR analysis. The PCR reaction contained 10 μL of 2× PCR mix, 2 μL of genomic DNA, 0.5 μL of each of the upstream and downstream primers (10 μM), and 7 μL ddH_2_O. The PCR reaction proceeded as follows: 3 min of pre-denaturation at 94 °C, followed by 20 s of denaturation at 94 °C, 20 s of annealing at 56 °C, and 30 s of extension at 72 °C (35 cycles for last three steps). Finally, there was 5 min of final extension at 72 °C and eternal storage at 4 °C.

### 2.8. Establishment of One-Pot RAA-dsDNase-UV and One-Pot RAA-dsDNase-LFD Detection Methods

Once the RAA-dsDNase specificity and sensitivity analysis is finished, the experiment will be made simpler and the chance of aerosol contamination from frequent lid opening and shutting will be decreased. In this investigation, an EP tube was concurrently filled with two RAA and dsDNase reaction systems. The RAA reaction system was positioned at the tube’s bottom, and the cap was filled with dsDNase, ssDNase probes, and ddH_2_O. Following the completion of the RAA reaction, a quick centrifugation was carried out, and the tube was turned upside down to thoroughly mix the two reaction systems. After that, incubation was carried out for 10 min at 37 °C. The outcomes were then identified as one-pot RAA-dsDNase-UV and one-pot RAA-dsDNase-LFD, respectively.

### 2.9. Sensitivity and Specificity Analysis of One-Pot RAA-dsDNase-UV and One-Pot RAA-dsDNase-LFD

In addition to adding the two reaction systems of RAA and dsDNase separately to the bottom and top of the EP tube, the sensitivity and specificity analysis process of one pot of RAA-dsDNase-UV and one pot of RAA-dsDNase-LFD was the same as steps outlined above. In addition, the two reaction systems of RAA and dsDNase separately were added to the bottom and top of the EP tube. Additionally, the 3′ modification of probe-2 was changed to biotin.

### 2.10. Detection of Salmonella in Real Samples

In order to test the practicality of one pot of RAA-dsDNase-UV and one pot of RAA-dsDNase-LFD in suspected *Salmonella* samples, we dissected clinically suspected *Salmonella* pullorum-infected chicks and retrieved tissue DNA from their organs, including the heart, liver, spleen, lungs, kidneys, and intestinal tissue. The main symptoms of chicks infected with chicken pullorum disease include lethargy, curling up, wings drooping, anorexia, and excretion of white viscous diarrhea. The necropsy results of sick chickens showed that the livers of these animals were enlarged, blackened, fragile, and accompanied by severe nodules. Then, we conducted analyses on *Salmonella* utilizing PCR, one-pot RAA-dsDNase-UV, one-pot RAA-dsDNase-LFD, and conventional bacterial isolation and culture techniques.

To further evaluate the practicality of one-pot RAA-dsDNase-UV and one-pot RAA-dsDNase-LFD in food, we purchased commercial milk from a local supermarket and confirmed its freedom from *Salmonella* contamination through the plate counting method. Then, we added *Salmonella* serotypes such as *S. enteritidis*, *S. thompson*, *S. typhimurium*, *S. derby*, and *S. infantis* to the milk to simulate the natural contamination of *Salmonella* in milk, with a final concentration of 1 × 10^6^ CFU/mL. Subsequently, bacterial genomic DNA was extracted and analyzed by one-pot RAA-dsDNase-UV and one-pot RAA-dsDNase-LFD. Throughout the process, milk without *Salmonella* contamination was selected as a control.

## 3. Results

### 3.1. Design and Screening of RAA Primers and dsDNAse Probes

For the highly conserved sequences of the *hilA* gene in the NCBI database, nine pairs of RAA primers, one pair of PCR primers, and two ssDNA fluorescent probes were designed (Table 1). The electrophoresis results demonstrated that the amplification band corresponding to primer F7R7 was single and the brightest when the same quality of template DNA and consistent reaction conditions were used (Figure S2). Probe screening results revealed that there was no fluorescence in the control group and that the fluorescence intensity of the probe-2 group was much higher than that of the probe-1 group (Figure S3). For the purpose of the next experimental study, primer pair F7R7 and probe-2 were employed.

### 3.2. RAA-dsDNase Principle and Feasibility Analysis

**Figure 1:**
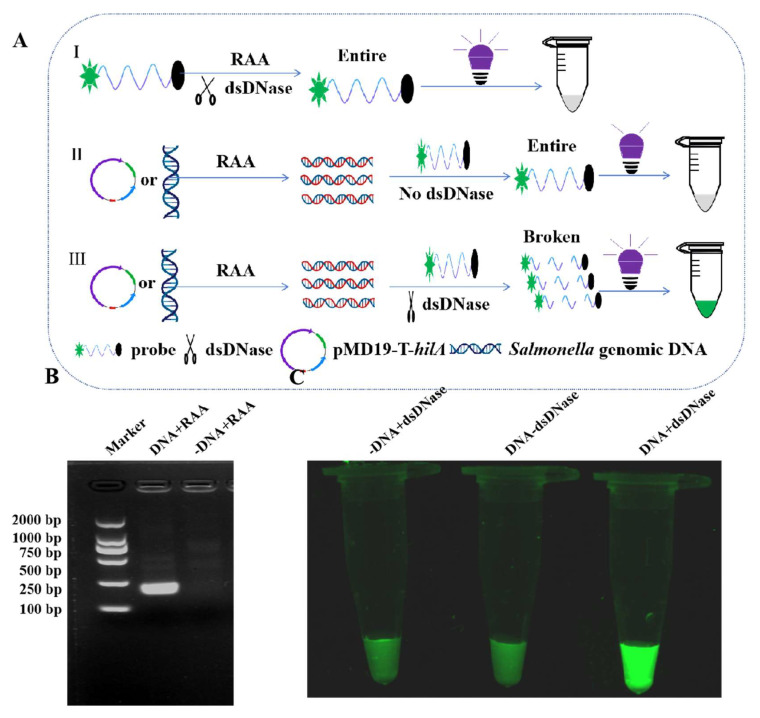
Schematic diagram and feasibility analysis of RAA-dsDNase detection for *Salmonella*. (**A**) The principle of RAA-dsDNase detection for *Salmonella* mainly includes three parts. (**I**) dsDNase has no degrading activity on ssDNA fluorescent probe. (**II**) When dsDNase was absent from the reaction system, the ssDNA fluorescent probe remains intact. (**III**) When the template DNA was amplified by RAA, then dsDNase was added to the system, and the ssDNA fluorescent probe was degraded and released a fluorescent signal. (**B**,**C**) Feasibility analysis of RAA-dsDNase detection for *Salmonella*.

The principle of detecting *Salmonella* by RAA-dsDNase method lies in massively amplifying *Salmonella*-specific genes using RAA technology. The fluorescent signal is released when the dsDNase breaks down the ssDNA fluorescent probe after it binds to the target region to produce a double strand (Figure 1A). We discovered that the RAA method worked well for large-scale gene amplification through feasibility analysis (Figure 1B). We then used dsDNase to analyze the degradation of ssDNA fluorescent probes. The findings demonstrated that in the absence of template DNA or dsDNase in the reaction system, fluorescence signals could not be seen (Figure 1C).

### 3.3. Optimization of Reaction Buffer

The reaction buffer must be optimized because variables in the reaction system, such as the concentration of magnesium ions, may have an impact on the activity of dsDNase. Amounts of 0, 0.5 μL, and 1 μL of 10× dsDNase buffer were added individually to the dsDNase reaction system. The findings demonstrated that there was no discernible variation in fluorescence intensity between the reaction systems, suggesting that the RAA-dsDNase reaction is not significantly impacted by 10× dsDNase buffer (Figure S4). As a result, it was not included in the RAA-dsDNase reaction that followed. This is primarily because all the ingredients needed for the dsDNase reaction are present in the RAA reaction buffer.

### 3.4. Optimization of dsDNase Dosage

The dsDNase activity is directly influenced by its dosage. Therefore, to maximize its maximum reaction activity and control the cost, we need to optimize the dosage of dsDNase. When dsDNase was at 1 μL, fluorescence intensity in the reaction system was significantly higher compared to 0.5 μL and 0.75 μL, suggesting that 1 μL of dsDNase is suitable for experiments (Figure S5).

### 3.5. Optimization of Reaction Time

Fluorescence probe cleavage may be incomplete if the dsDNase reaction time is too short; on the other hand, an excessive reaction time reduces detection efficiency. As a result, we ran our studies for three different durations: 0 min, 5 min, and 10 min. As a result, and as Figure S6 illustrates, fluorescence signals were strong at 5 and 10 min, with a small rise at the latter time. The 10 min reaction time was chosen for the following tests in order to guarantee the sensitivity of RAA-dsDNase.

### 3.6. Sensitivity and Specificity Analysis of RAA-dsDNase and PCR Specificity Analysis

**Figure 2:**
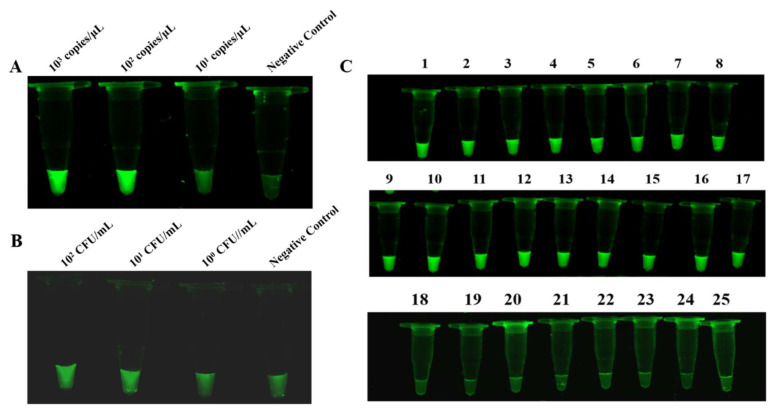
Sensitivity and specificity analysis of RAA-dsDNase. (**A**,**B**) The detection limit of RAA-dsDNase was 101 copies/μL (plasmid) and 101 CFU/mL (bacteria). (**C**) When the genomic DNA of *Salmonella* exists, RAA-dsDNase releases a strong fluorescent signal, while in the presence of genomic DNA of other species, including *Escherichia coli*, no fluorescent signal was produced. Strain numbers correspond to the details in Table S1.

Sensitivity investigation showed that the RAA-dsDNase reaction system exhibited strong fluorescence within the plasmids concentration range of 10^2^ to 10^3^ copies/μL. The fluorescence signal was modest but identifiable from the control group even at a plasmid concentration of 10^1^ copies/μL (Figure 2A). RAA-dsDNase in bacterial genome testing revealed that, in comparison to 0 and 10^0^ CFU/mL, the fluorescence intensity of bacteria with 10^2^ and 10^1^ CFUs was much higher (Figure 2B). Nevertheless, a discernible variation in fluorescence intensity between 0 and 10^1^ CFU/mL is not visible to the unaided eye. The findings of the sensitivity investigation showed that the detection limit of RAA-dsDNase is 10^1^ CFU/mL (bacteria) and 10^1^ copies/μL (plasmid).

The RAA-dsDNase reaction system produced a significant fluorescence signal when *Salmonella* genomic DNA (Figure 2C, nos. 1–17) was used as the template, according to specificity analysis. No cross-reaction was seen between the other eight strains, including E. coli (Figure 2C, nos. 18–25). Similar results were obtained from PCR analysis; bands were generated in agarose gel electrophoresis only in the presence of *Salmonella* genomic DNA, while no bands were observed in other bacterial genomic DNA (Figure S7). Table S1 displays the strains that correspond to numbers 1 through 25. This supports the great specificity of the RAA-dsDNase results.

### 3.7. Establishment of One-Pot RAA-dsDNase-UV and One-Pot RAA-dsDNase-LFD Detection Methods

**Figure 3:**
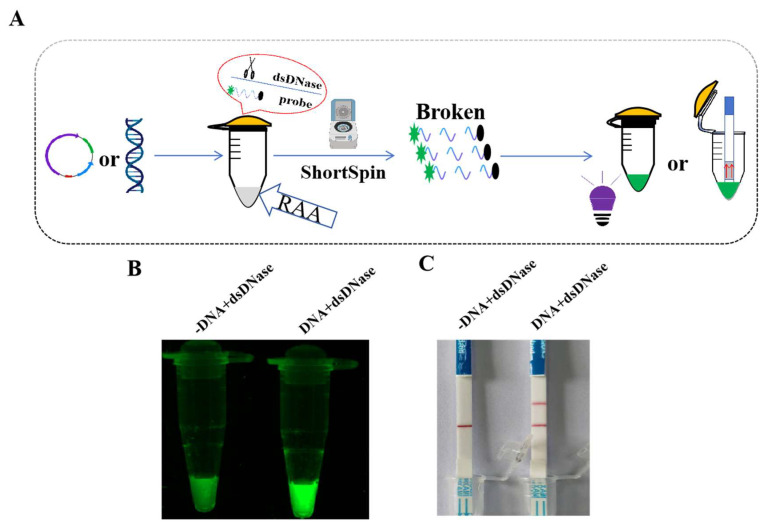
Schematic diagram and feasibility analysis of one-pot RAA-dsDNase-UV and one-pot RAA-dsDNase-LFD detection for *Salmonella*. (**A**) Simultaneously adding the components of the RAA and dsDNase reactions into an EP tube, with RAA at the bottom of the tube and dsDNase and the fluorescent probe at the tube cap. After the RAA reaction was completed, it was briefly centrifuged, inverted to mix, and finally the results were observed using UV and LFD. (**B**,**C**) The feasibility analysis results showed that fluorescence or detection bands appeared only when the genomic DNA of *Salmonella* existed.

We conducted a one-pot RAA-dsDNase experiment, which reduced the possibility of contamination and simplified the reaction process despite the high specificity and sensitivity of RAA-dsDNase. The findings were visualized using UV and LFD. The schematic diagram for a one-pot process is shown in Figure 3A. This method involved adding the fluorescent probe, ddH_2_O, and dsDNase to the tube lid after the RAA mixture was positioned at the tube’s bottom. Fluorescence or detection bands were only created in EP tubes containing DNA templates, as shown in Figure 3B,C. This indicates that the one-pot RAA-dsDNase-UV and one-pot RAA-dsDNase-LFD procedures are feasible.

### 3.8. Sensitivity and Specificity Analysis of One-Pot RAA-dsDNase-UV and One-Pot RAA-dsDNase-LFD

**Figure 4:**
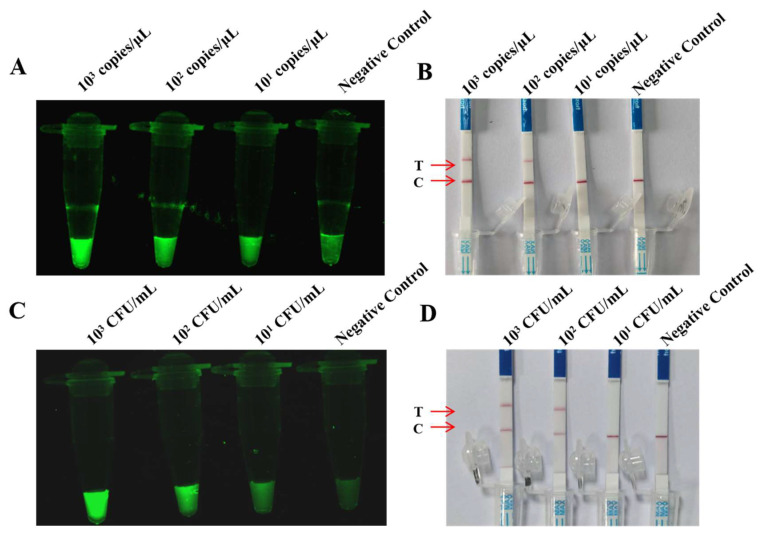
Sensitivity analysis of one-pot RAA-dsDNase-UV and one-pot RAA-dsDNase-LFD. (**A**,**C**) The detection limit of one-pot RAA-dsDNase-UV was 101 copies/μL (plasmid) and 101 CFUs (bacteria). (**B**,**D**) The detection limit of one-pot RAA-dsDNase-LFD was 102 copies/μL (plasmid) and 102 CFU/mL (bacteria).

**Figure 5:**
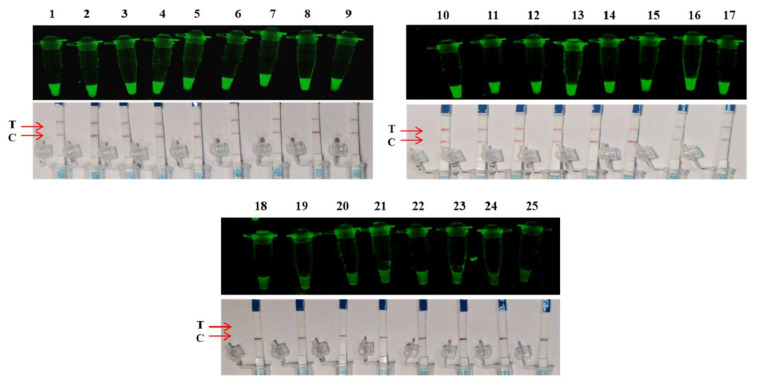
Specificity analysis of one-pot RAA-dsDNase-UV and one-pot RAA-dsDNase-LFD. Specificity analysis results showed that one-pot RAA-dsDNase-UV and one-pot RAA-dsDNase-LFD could specifically detect *Salmonella*, without cross-reactivity to other strains of bacteria. Strain numbers correspond to the details in Table S1.

The detection limit of one-pot RAA-dsDNase-UV was determined by sensitivity analysis to be 10^1^ copies/μL for plasmids and 10^1^ CFU/mL for bacteria (Figure 4A,B). Additionally, Figure 4C,D show that the one-pot RAA-dsDNase-LFD detection limits were 10^2^ copies/μL for plasmids and 10^2^ CFU/mL for bacteria. The results of the specificity investigation were in perfect agreement with RAA-dsDNase (Figure 5).

### 3.9. Results of Salmonella Analysis in Tissues Using One-Pot RAA-dsDNase-UV and One-Pot RAA-dsDNase-LFD

**Figure 6:**
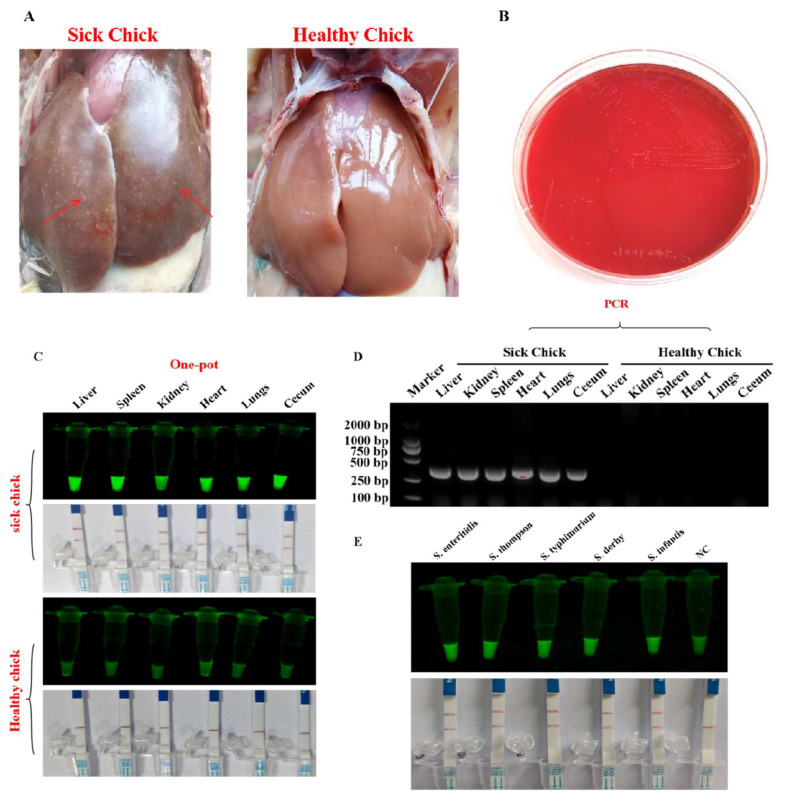
Detection of *Salmonella* in real samples. (**A**) Observation of liver tissue samples from sick and healthy chickens in clinical practice with the naked eye. The red arrow indicates the location of the nodule. (**B**) Traditional culture method was used to analyze *Salmonella* in the tissues of sick chickens, and the results showed typical morphology of *Salmonella* pullorum. (**C**) One-pot RAA-dsDNase-UV, one-pot RAA-dsDNase-LFD, and PCR analysis showed that significant fluorescent signals or detection bands were produced in the tissues of the hearts, livers, spleens, lungs, kidneys, and intestines of sick chicks, while no corresponding phenomenon was observed in healthy chicken tissues. (**D**) And similar results were obtained from PCR analysis. (**E**) Detection of *Salmonella* in milk using one-pot RAA-dsDNase-UV and one-pot RAA-dsDNase-LFD showed that both methods produced clear fluorescent signals or detection bands.

Suspected tissue samples of chickens infected with *Salmonella pullorum* were evaluated using one-pot RAA-dsDNase-UV and one-pot RAA-dsDNase-LFD, PCR, and conventional bacterial isolation culture after specificity and sensitivity tests were finished. The clinical autopsy of dead chickens shows that their livers are more fragile, with many nodules on the surface and a noticeably darker color (Figure 6A). Bacterial isolation and culture results showed that the isolated strain conforms to the typical morphology of *Salmonella* pullorum on XLT-4 agar medium (Figure 6B). A substantial fluorescent signal was obtained when ill chicken tissue samples were analyzed using one-pot RAA-dsDNase-UV and one-pot RAA-dsDNase-LFD. On the other hand, when applied to tissue samples from healthy chickens, neither technique produced any fluorescence (Figure 6C). When PCR and agarose gel electrophoresis were used to examine the tissue samples of sick hens, specific bands were seen; however, no discernible bands were seen in the samples from healthy chickens (Figure 6D).

### 3.10. Results of Salmonella Analysis in Milk Using One-Pot RAA-dsDNase-UV and One-Pot RAA-dsDNase-LFD

The detection results of *Salmonella* in milk using one-pot RAA-dsDNase-UV and one-pot RAA-dsDNase-LFD showed that when *Salmonella* contamination was present in the milk, both methods produced clear fluorescent signals or detection bands, indicating that one-pot RAA-dsDNase-UV and one-pot RAA-dsDNase-LFD can be used for the detection of *Salmonella* in food (Figure 6E).

## 4. Discussion

*Salmonella* is a highly prevalent food-borne pathogen that can cause poisoning in both humans and animals, which can lead to the spread of drug resistance and result in serious economic losses. This has become a global public health problem [27,28,29]. Currently, over 2600 serovars of *Salmonella* have been reported [30], which poses a significant challenge to the prevention and control of *Salmonella* owing to the large number of serotypes and the extensive detection efforts required. In recent years, DNA molecular detection techniques such as PCR (RT-PCR) [31], LAMP [32], RPA [33], and CRISPR/Cas [34] have been widely developed for *Salmonella* diagnosis. However, these methods have some limitations, such as the need for expensive equipment, professional personnel, complex primer designs, and the participation of RNA in the detection process. Therefore, the development of a simple, visible process that does not require high environmental requirements and can detect multiple *Salmonella* serotypes is of great significance for *Salmonella* prevention and control.

The *hilA* gene, a virulence regulator that plays a crucial role in the regulation of SPI-1, is a unique feature of *Salmonella* species and is absent in other Gram-negative bacteria. The up-regulation of this gene has been linked to enhanced colonization or organ invasion [25], and some studies have utilized *Salmonella* enteritidis lacking the *hilA* gene to create attenuated vaccine strains [35]. Previous studies have shown that PCR detection methods targeting the *hilA* gene have high specificity for *Salmonella* [36]. Another study successfully differentiated 83 different serovars of *Salmonella* and 22 non-*Salmonella* strains by targeting the *hilA* gene. A study that evaluated the suitability of targeting different genes (agfA, sef, spvC, and *hilA*) for *salmonella* detection showed that PCR methods targeting the *hilA* gene were all positive for *Salmonella* [37]. Based on these findings, it can be inferred that *hilA* serves as a highly specific target for *Salmonella* detection.

This work provided a new approach for detecting *Salmonella* by combining RAA with dsDNase. Large-scale *Salmonella hilA* gene amplification was accomplished quickly and effectively using RAA technology. Remarkably, a fluorescent signal was then released when the fluorescent probe and target complex were broken up by dsDNase. In order to establish visual one-pot RAA-dsDNase-UV and one-pot RAA-dsDNase-LFD, the results were seen under UV and LFD. One-pot RAA-dsDNase-UV and one-pot RAA-dsDNase-LFD had detection limits for *Salmonella* DNA of 10^1^ CFU/mL (50 min) and 10^2^ CFU/mL (60 min), respectively. The technique for detecting *Salmonella* that was developed in this work can be used to identify *Salmonella* without causing cross-reactivity with other strains, such as *E. coli*. Additionally, they have been effective in identifying *Salmonella* in chick tissue samples and milk.

Numerous techniques for identifying *Salmonella* have been developed as research advances. In this study, we compared the detection method developed by us with other *Salmonella* detection methods by referring to the expression method of Li et al. [38] (Table S2). Traditional cultivation methods are regarded as the gold standard for *Salmonella* detection, yet they are cumbersome, time-consuming, and have limited sensitivity [39,40]. The ELISA is a commonly used approach for detecting *Salmonella*; however, the manufacture of its antibody is rather challenging [41,42]. The PCR/RT-PCR technique is simple and effective, but it requires expensive equipment and is prone to contamination [43,44]. Although LAMP for *Salmonella* detection has an easy-to-use interface and effective amplification, it has drawbacks such as complicated primer construction and the possibility of false positives [45]. Simpleness, quick detection, and high specificity are among the advantages of CRISPR/Cas12a or Cas13a. However, PAM site limits in sgRNA design, strict sample processing requirements, and false positives/negatives make it faulty [46,47,48,49]. In comparison to the other approaches, RAA-dsDNase provides simplicity, speed, sensitivity, cost-effectiveness, and PAM site versatility, making it a viable option for *Salmonella* detection.

In conclusion, this study targeted the *Salmonella hliA* gene and constructed a rapid, highly sensitive, and specific one-pot RAA-dsDNase-UV and one-pot RAA-dsDNase-LFD *Salmonella* detection strategy based on RAA combined with dsDNase for the first time.

## Figures and Tables

**Table 1:** The nucleic acid sequences used in this study.

Name	Sequences (5′–3′)
RAA-F1	AGGATATTCTTGAGCTCATGGATCAATTACG
RAA-R1	CAATTTTGTTTTGCAAGAGAGAAGCGGGTT
RAA-F2	AGCTCATGGATCAATTACGCCCCGATTATT
RAA-R2	CAAAAGATTCGCAATTTTGTTTTGCAAGAG
RAA-F3	ATTATTATATCTCCGGGCAGATGATACCCGAT
RAA-R3	TTTGTGTCCCAGCGAAGTCCGGGAATACAT
RAA-F4	ATTATTATATCTCCGGGCAGATGATACCCGAT
RAA-R4	CAAAAGATTCGCAATTTTGTTTTGCAAGAG
RAA-F5	GCAGATGATACCCGATGGTAATGATAATATTGT
RAA-R5	CGGGAATACATCTGAGCAAAAGATTCGCAA
RAA-F6	CCGGGCAGATGATACCCGATGGTAATGATA
RAA-R6	TTTGTGTCCCAGCGAAGTCCGGGAATACAT
RAA-F7	ATGGATCAATTACGCCCCGATTATTATATCTCC
RAA-R7	CGGGAATACATCTGAGCAAAAGATTCGCAA
RAA-F8	AGGATATTCTTGAGCTCATGGATCAATTACG
RAA-R8	TTTGTGTCCCAGCGAAGTCCGGGAATACAT
RAA-F9	AGCTCATGGATCAATTACGCCCCGATTATT
RAA-R9	GAAGTCCGGGAATACATCTGAGCAAAAGAT
PCR-F	GTGACCATTACGAAGAACTG
PCR-R	TGTTTGGCGACATGTTAAC
Probe-1	FAM-GTTAAAGGTTATCACC-BHQ1
Probe-2	FAM-GAAAGCATTAAGTTGA-BHQ1
Probe-2.1	FAM-GAAAGCATTAAGTTGA-Bio

## Data Availability

The original contributions presented in the study are included in the article/Supplementary Materials, further inquiries can be directed to the corresponding author.

## Supplementary Materials

The following supporting information can be downloaded at https://www.mdpi.com/article/10.3390/foods13091380/s1, Figure S1: pMD19-T-*hilA* plasmid map; Figure S2: RAA primer screening. Amplification of the target hilA gene using RAA under the same conditions, followed by analysis of the amplification products using 2% agarose gel electrophoresis; Figure S3: ssDNA fluorescent probe screening screening. Fluorescent probe screening: Probe 1 and Probe 2 were added to the reaction system, and it was observed that Probe 2 exhibited significantly stronger fluorescence than Probe 1 under UV light; Figure S4: Optimization of reaction buffer. 0, 0.5 μL, and 1 μL of 10x dsDNase buffer were added to the reaction system, and no significant difference in fluorescence intensity was observed under UV light; Figure S5: Optimization of dsDNase dosage. 0.5 μL, 0.75 μL, and 1 μL of dsDNase were added to the reaction system, and the maximum fluorescence intensity was observed when 1 μL of dsDNase was added; Figure S6: Optimization of reaction time. Fluorescence intensity is significantly higher at 5 minutes and 10 minutes after dsDNase treatment compared to 0 minutes, reaching the highest value at 10 minutes; Figure S7: PCR detection of *Salmonella* and other strains. Strain numbers correspond to the details in Table S1; Table S1: Bacteria strains used in this study. Table S2: Comparation of the RAA-dsDNase with other assays for *Salmonella* detecting. Refs. [50,51,52,53] are cited in Supplementary Materials.
