# Supplementary material for: Establishment of a Simple, Sensitive, and Specific Salmonella Detection Method Based on Recombinase-Aided Amplification Combined with dsDNA-Specific Nucleases
Source: Foods. 2024 Apr 29;13(9):1380. doi: 10.3390/foods13091380 (PMC11083397; doi:10.3390/foods13091380)
Supplement: Supplementary file 1 [file foods-13-01380-s001.zip › foods-2968606-supplementary.pdf]

Supplementary Material

Establishment of a Simple, Sensitive, and Specific *Salmonella* Detection Method Based on Recombinase-Aided Amplification Combined with dsDNA-Specific Nucleases

Changyu Zhou [1,2,†], Yu Zhao [1,2,†], Boyan Guo [1,2], Ming Yang [1,2], Qiang Xu [1,2], Changwei Lei [1,2] and Hongning Wang [1,2,*]

[1] Key Laboratory of Bio-Resource and Eco-Environment of Ministry of Education, College of Life Sciences, Sichuan University, Chengdu 610017, China; l1z2l3y4@163.com (C.Z.); zhaoyu3@stu.scu.edu.cn (Y.Z.); gby0229@163.com (B.G.)

[2] Animal Disease Prevention and Food Safety Key Laboratory of Sichuan Province, Chengdu 610064, China

* Correspondence: hongningwang@scu.edu.cn

† These authors contributed equally to this work and considered as co-first authors.

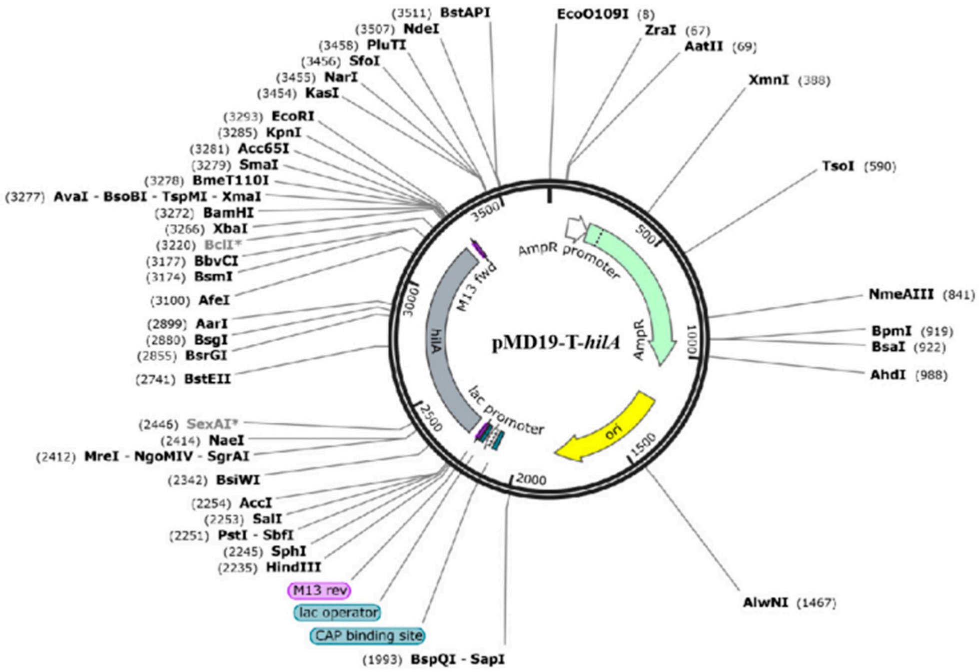

Figure S1. pMD19-T-*hilA* plasmid map

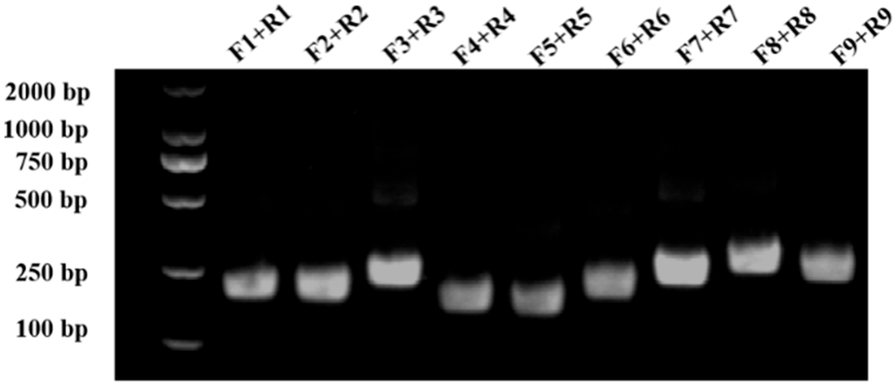

Figure S2. RAA primer screening. Amplification of the target hilA gene using RAA under the same conditions, followed by analysis of the amplification products using 2% agarose gel electrophoresis.

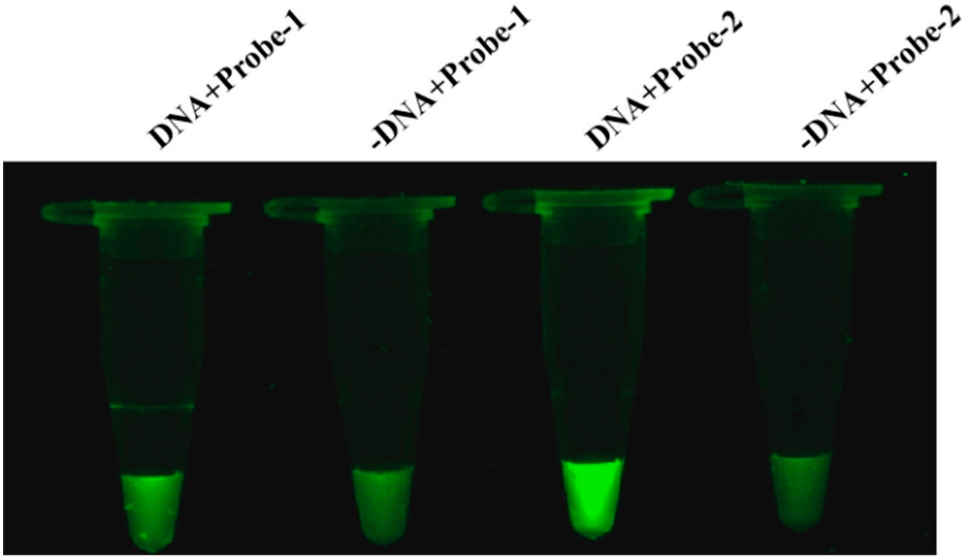

Figure S3. ssDNA fluorescent probe screening screening. Fluorescent probe screening: Probe 1 and Probe 2 were added to the reaction system, and it was observed that Probe 2 exhibited significantly stronger fluorescence than Probe 1 under UV light.

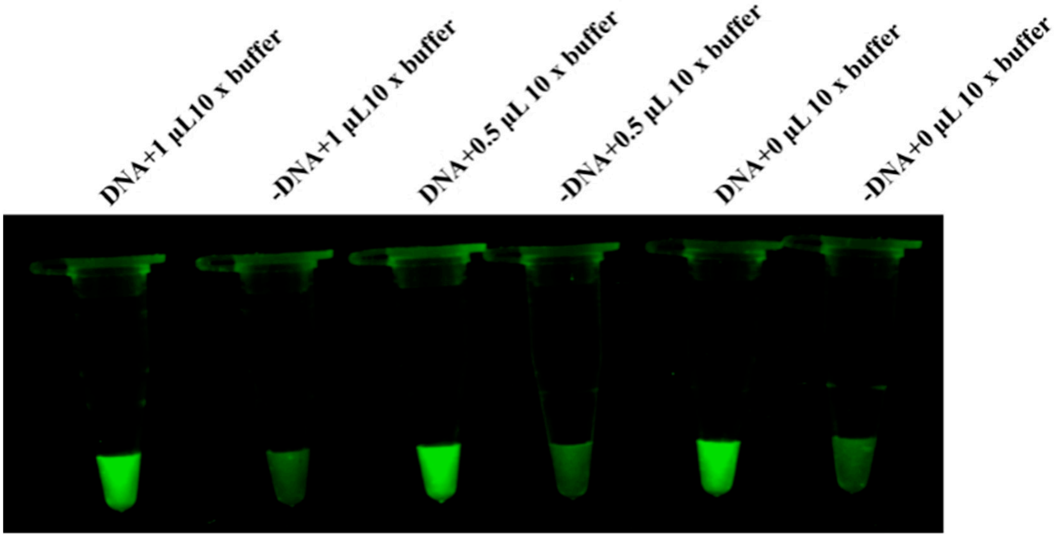

Figure S4. Optimization of reaction buffer. 0, 0.5 μL, and 1 μL of 10x dsDNase buffer were added to the reaction system, and no significant difference in fluorescence intensity was observed under UV light.

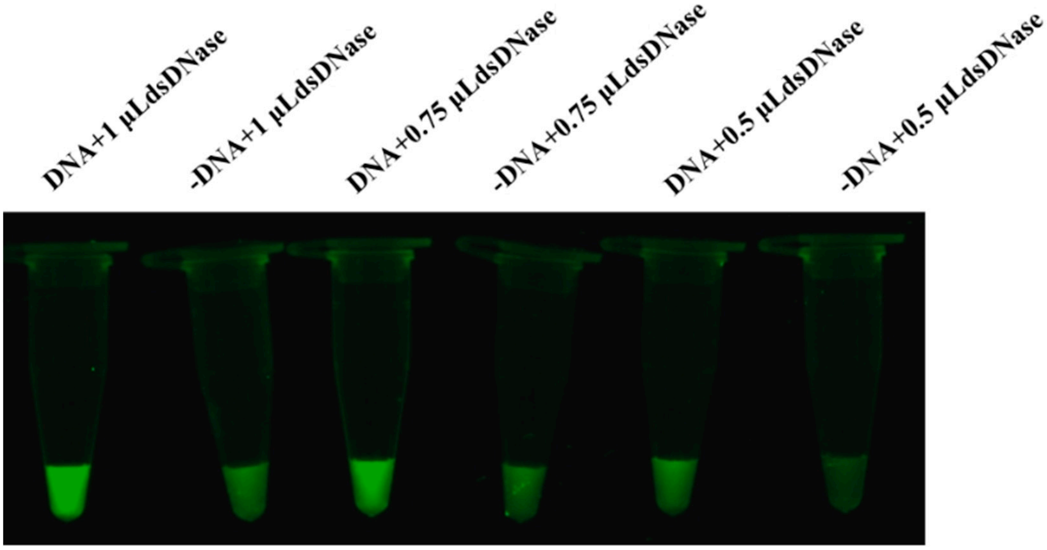

Figure S5. Optimization of dsDNase dosage. 0.5 μL, 0.75 μL, and 1 μL of dsDNase were added to the reaction system, and the maximum fluorescence intensity was observed when 1 μL of dsDNase was added.

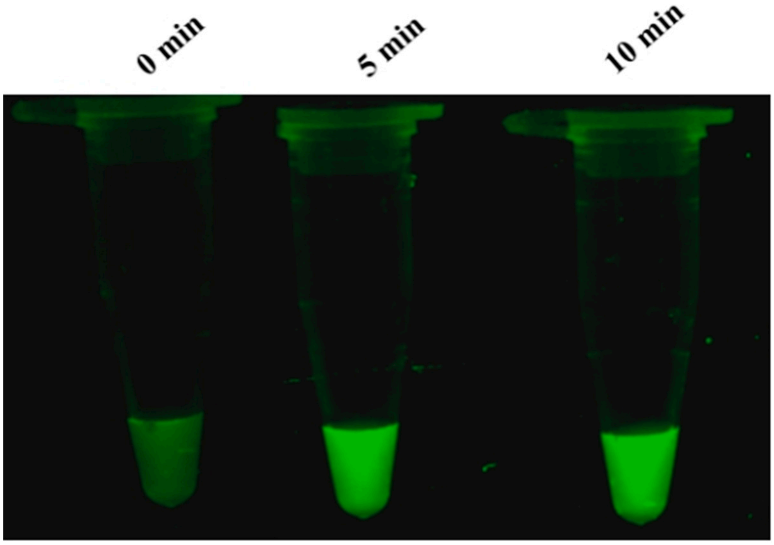

Figure S6. Optimization of reaction time. Fluorescence intensity is significantly higher at 5 minutes and 10 minutes after dsDNase treatment compared to 0 minutes, reaching the highest value at 10 minutes.

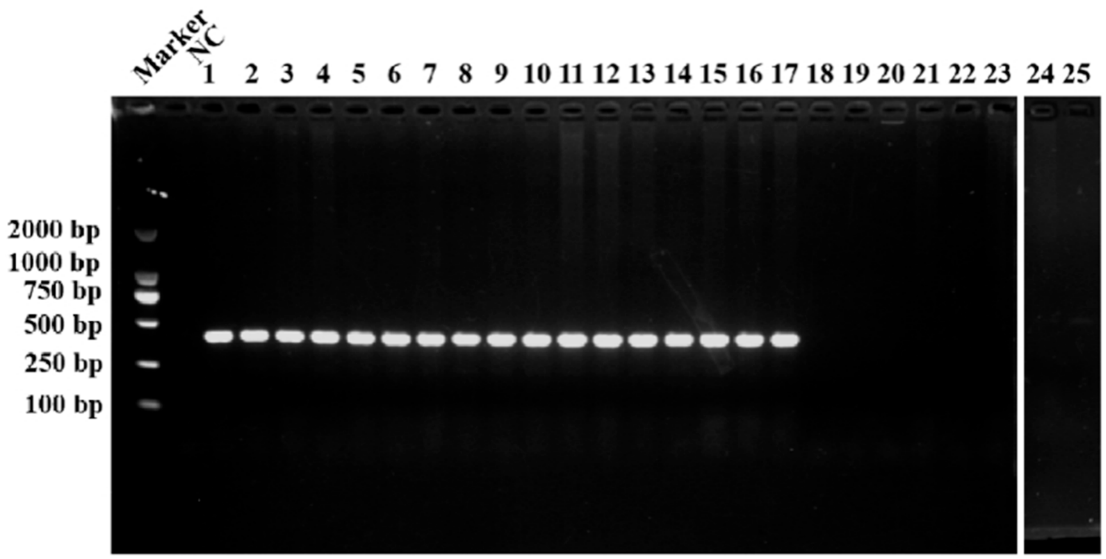

Figure S7. PCR detection of Salmonella and other strains. Strain numbers correspond to the details in Table S1.

Table S1. Bacteria strains used in this study.

Number*	Strain types**
1	Salmonella Enteritidis
2	Salmonella Indiana
3	Salmonella Typhimurium
4	Salmonella Thompson
5	Salmonella Infantis
6	Salmonella potential monophasic variant of Typhimurium
7	Salmonella Derby
8	Salmonella Anatum
9	Salmonella Schwarzengrund
10	Salmonella London
11	Salmonella Kentucky
12	Salmonella Mbandaka
13	Salmonella Goldcoast
14	Salmonella Meleagridis
15	Salmonella Rissen
16	Salmonella Give
17	Salmonella pullorum
18	Klebsiella pneumoniae
19	Escherichia coli
20	Proteus mirabilis
21	Pseudomonas aeruginosa
22	Acinetobacter baumannii
23	Cronobacter sakazakii
24	Staphylococcus aureus
25	Enterococcus faecalis

* Specificity test experiment corresponding number. **All strains were maintained in the Animal Disease Prevention and Food Safety Key Laboratory of Sichuan Province, Sichuan University.

Table S2. Comparation of the RAA-dsDNase with other assays for Salmonella detecting.

Methods	Pathogens	Strategy	LOD (CFU/mL)	Reference
Culture methods		Bacteria	1 CFU/g	ISO 6579-1:2017
PCR	Salmonella	DNA	6.7×10^3	[8]
qPCR	S. Enteritidis	DNA	40	[13]
Surface plasmon resonance	S. Typhimurium		10^4	[50]
Immunoassays	S. Enteritidis	Bacteria	5×10^4	[51]
LAMP	Salmonella	DNA	6.7	[8]
Electrochemical biosensor	S. Typhimurium	bacteria	2	[52]
Gas-driven capillary	S. Typhimurium	DNA	37	[53]
CRISPR/Cas12a and silver nanoclusters	S. Typhimurium	DNA	1	[46]
RPA-Cas13a	Salmonella	DNA	10^2 copies	[14]
RAA-dsDNase	Salmonella	DNA	10	This work

Reference

[8] X. Mei, X. Zhai, C. Lei, X. Ye, Z. Kang, X. Wu, R. Xiang, Y. Wang, H. Wang, Development and application of a visual loop-mediated isothermal amplification combined with lateral flow dipstick (LAMP-LFD) method for rapid detection of Salmonella strains in food samples, Food Control, 104 (2019) 9-19.
[13] J. Shen, X. Zhou, Y. Shan, H. Yue, R. Huang, J. Hu, D. Xing, Sensitive detection of a bacterial pathogen using allosteric probe-initiated catalysis and CRISPR-Cas13a amplification reaction, Nature communications, 11 (2020) 267.
[50] S.H. Hyeon, W.K. Lim, H.J. Shin, Novel surface plasmon resonance biosensor that uses full-length Det7 phage tail protein for rapid and selective detection of Salmonella enterica serovar Typhimurium, Biotechnology and applied biochemistry, 68 (2021) 5-12.
[51] K. Gu, Z. Song, C. Zhou, P. Ma, C. Li, Q. Lu, Z. Liao, Z. Huang, Y. Tang, H. Li, Y. Zhao, W. Yan, C. Lei, H. Wang, Development of nanobody-horseradish peroxidase-based sandwich ELISA to detect Salmonella Enteritidis in milk and in vivo colonization in chicken, Journal of nanobiotechnology, 20 (2022) 167.
[52] Y. Ding, Y. Zhang, C. Huang, J. Wang, H. Li, X. Wang, An electrochemical biosensor based on phage-encoded protein RBP 41 for rapid and sensitive detection of Salmonella, Talanta, 270 (2024) 125561.
[53] L. Gao, Y. Ge, J. Xie, Y. Li, H. Zhang, S. Du, A gas-driven capillary based on the synergy of the catalytic and photothermal effect of PB@Au for Salmonella typhimurium detection, Talanta, 269 (2024) 125455.
[46] L. Ma, J. Wang, Y. Li, D. Liao, W. Zhang, X. Han, S. Man, A ratiometric fluorescent biosensing platform for ultrasensitive detection of Salmonella typhimurium via CRISPR/Cas12a and silver nanoclusters, Journal of hazardous materials, 443 (2023) 130234.
[14] B. An, H. Zhang, X. Su, Y. Guo, T. Wu, Y. Ge, F. Zhu, L. Cui, Rapid and Sensitive Detection of Salmonella spp. Using CRISPR-Cas13a Combined With Recombinase Polymerase Amplification, Frontiers in microbiology, 12 (2021) 732426.
